# Determination of polycyclic aromatic hydrocarbons (PAHs) and other organic pollutants in freshwaters on the western shore of Admiralty Bay (King George Island, Maritime Antarctica)

**DOI:** 10.1007/s11356-019-05045-w

**Published:** 2019-04-29

**Authors:** Małgorzata Szopińska, Danuta Szumińska, Robert Józef Bialik, Tomasz Dymerski, Erwin Rosenberg, Żaneta Polkowska

**Affiliations:** 1 Faculty of Civil and Environmental Engineering, Department of Water and Waste Water Technology, Gdansk University of Technology, 11/12 Narutowicza St., 80-233 Gdansk, Poland; 2 Institute of Geography, Kazimierz Wielki University, 8 Kościelecki Sq., 85-033 Bydgoszcz, Poland; 3 Polish Academy of Science, Institute of Biochemistry and Biophysics, Pawińskiego 5a, 02-106 Warsaw, Poland; 4 Faculty of Chemistry, Department of Analytical Chemistry, Gdansk University of Technology, 11/12 Narutowicza St., 80-233 Gdańsk, Poland; 5 Institute of Chemical Technologies and Analytics, Vienna University of Technology, Getreidemarkt 9/164 AC, A-1060 Vienna, Austria

**Keywords:** Admiralty Bay, Arctowski Polish Antarctic Station, Freshwater chemistry, Maritime Antarctica, Organic pollution, Polycyclic aromatic hydrocarbons (PAHs)

## Abstract

Organic contamination in freshwater samples has never been investigated at the western shore of Admiralty Bay. Therefore, the presence of polycyclic aromatic hydrocarbons (PAHs) in five different sites distributed along a shore running from the Arctowski Station to the Baranowski Glacier was studied. Moreover, organic compounds such as n-alkanes, toluene and ethylbenzene were also noted. Increased ΣPAHs in late Austral summer 2016 are the result of long-range atmospheric transport of air masses from South America, confirmed by 10-day backward air mass trajectories analysis. The presence of n-alkanes and other hydrocarbons, as well as the evaluation of PAH indices (e.g. ΣLMW/ΣHMW* > 1), shows the use of fuel and indicate local human activity. As a final conclusion, our analysis indicates a mixed origin of PAHs (global and local). The presence of PAHs and other hydrocarbons in the water environment may constitute a potentially negative effect on the Antarctic ecosystem and it should be investigated in detail during further research (//*ΣLMW—sum of low molecular weight PAHs (two- and three-ring PAHs); ΣHMW—sum of high molecular weight PAHs (four- and five-ring PAHs)//).

**Electronic supplementary material:**

The online version of this article (10.1007/s11356-019-05045-w) contains supplementary material, which is available to authorized users.

**Figure:**
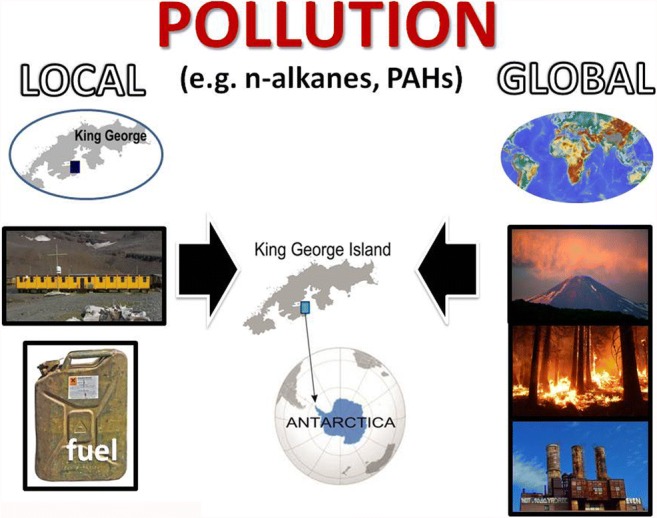
Graphical abstract

## Introduction

Antarctic ecosystems have been subject to increased human pressure for at least the past six decades (Bargagli 2008; Corsolini 2009; Szopińska et al. 2017). The map of selected persistent organic pollutants (POPs) in biotic and abiotic samples shows the presence of a broad range of chemical compounds related to human activity in various elements of the Antarctic environment (Potapowicz et al. 2019). The authors indicated the growing local human influence, of the presence of selected contaminants, e.g. polycyclic aromatic hydrocarbons (PAHs) (Cripps 1992; Martins et al. 2010), heavy metals (Santos et al. 2005; Mão de Ferro et al. 2013; Amaro et al. 2015) and polybrominated diphenyl ethers (PBDEs) (Hale et al. 2008). Moreover, presence of some compounds such as polychlorinated biphenyls (PCBs) (Inomata et al. 1996; Montone et al. 2001, 2003), dichlorodifenylotrichloroetan (DDTs) (Montone et al. 2005, Cabrerizo et al. 2012) shows the global human influence, including long-range atmospheric transport of pollutants (Bicego et al. 2009; Bengtson Nash et al. 2011; Kallenborn et al. 2013). The presence of organic pollution in pristine areas is observed not only in Antarctica but also in other places considered as poles: in the Arctic (Kozak et al. 2013) and the Himalayas (Loewen et al. 2005).

Moreover, increasing human influence on Antarctica coexists with processes related to climate changes. The climate in the Antarctic Peninsula has been subject to one of the most rapid warming phases on Earth in recent decades (until the middle of the twentieth century (Vaughan et al. 2003; Turner et al. 2005; Bockheim et al. 2013). However, from the late twentieth century to the first decades of 21st cooling is observed (Oliva et al. 2017b, Turner et al. 2016). Nevertheless, rapid glacier retreat (Rückamp et al. 2011; Pętlicki et al. 2017, Pudełko et al. 2018) and high-intensity morphological processes have affected rates of erosion and sediment transport, and weathering processes (Oliva et al. 2016, 2017a; Navas et al. 2017). This may cause the release of pollution accumulated in sediments (Martins et al. 2010), ice (Herbert et al. 2006a) and permafrost (Curtosi et al. 2007). Herbert et al. (2006a, b) note that quantities of pollutants released during the spring snowmelt could have a significant influence on the quantities of pollutants present in both freshwater and marine systems.

Although the Protocol on Environmental Protection to the Antarctic Treaty establishes obligations for all human activity and provides strict guidelines for protection in the Antarctic, a couple of factors such as global warming, population growth and industrial development in countries of the Southern Hemisphere will cause the impact of anthropogenic contaminants on Antarctic ecosystems to increase (Bargagli 2008). Despite the importance of extending the knowledge about Antarctic contamination (Bargagli 2008; Szopińska et al. 2017; Corsolini 2009), there are many gaps in our knowledge regarding the potential long-term effects of the presence of contaminants in the Antarctic environment, especially surfactants, endocrine disrupting chemicals (e.g. phthalates), polychlorinated biphenyls (PCBs), pesticides and polycyclic aromatic hydrocarbons (PAHs). There is also a lack of long-term future monitoring plans and environmental conservation strategies for the polar regions with respect to POPs (Mangano et al. 2017). Furthermore, information is especially lacking in runoff water chemistry characteristics. Most publications in the last decade have focused on soil (22%), precipitation (22%), air (18%) and seawater (16%) samples. Studies of freshwater samples constitute only 7% of studied published research (Szopińska et al. 2017).

Besides growing anthropopressure and the observed climate changes, the other reasonable issue that is an important part of the contamination research in polar regions is the phenomenon of bioaccumulation. Due to their lipophilic properties, organic compounds may be bio-accumulated in biota (Hale et al. 2008) and living organism tissues (Montone et al. 2016), causing negative effects. Because some PAHs are harmful to the environment (Straif et al. 2006), it would seem important to pay special attention to this group of compounds in such a pristine area as the Antarctic. This selected group of contaminants represents a special threat to the Antarctic environment because of their toxic, mutagenic and (in some cases) carcinogenic properties (Stogiannidis and Laane 2015; Rengarajan et al. 2015). Hence, the main aim of this study was to determine compounds from the group of polycyclic aromatic hydrocarbons. In detail, the objectives of the present study were (1) to elucidate the current spatial distribution and component characteristic of PAHs via a survey of runoff water and (2) to identify potential sources of PAHs using congener ratios and principal component analysis together with backward air mass trajectory analysis. In addition, the variety of chemical compounds (volatile and semi-volatile) occurring in selected runoff water samples taken from the western shore of Admiralty Bay was characterised. Moreover, the majority of the study area belongs to the Antarctic Specially Protected Area, ASPA No. 128, which was established against unforeseen and potentially hazardous human activity. This area protects featuring of unusual assemblages of species, including penguin colonies, where all three Pygoscelis penguins (Adélie (*Pygoscelis adeliae*), Gentoo (*Pygoscelis papua*) and Chinstrap (*Pygoscelis antarcticus*)) are found breeding together at the same location, and mammals, e.g. elephant seals (*Mirounga leonina*), Antarctic fur seals (*Arctocephalus gazella*) and Weddell seals (*Leptonychotes weddellii*). Most of the Antarctic organisms do not have advanced detoxification mechanisms (Bengtson Nash et al. 2011), and even a relatively small amount of contamination may represent a threat to them. Thus, particular attention should be paid to monitoring actual organic contamination levels in the studied area of Maritime Antarctica. Furthermore, this type of study has never been conducted in ASPA No. 128 and its surroundings; hence, our findings will have great value for this local Antarctic environment. We hope that it will increase the actions taken to help preserve the diversity of the sensitive Antarctic ecosystem in this area, e.g. a general reduction in fuel consumption.

## Materials and method

### Sample collection and design

**Fig. 1:**
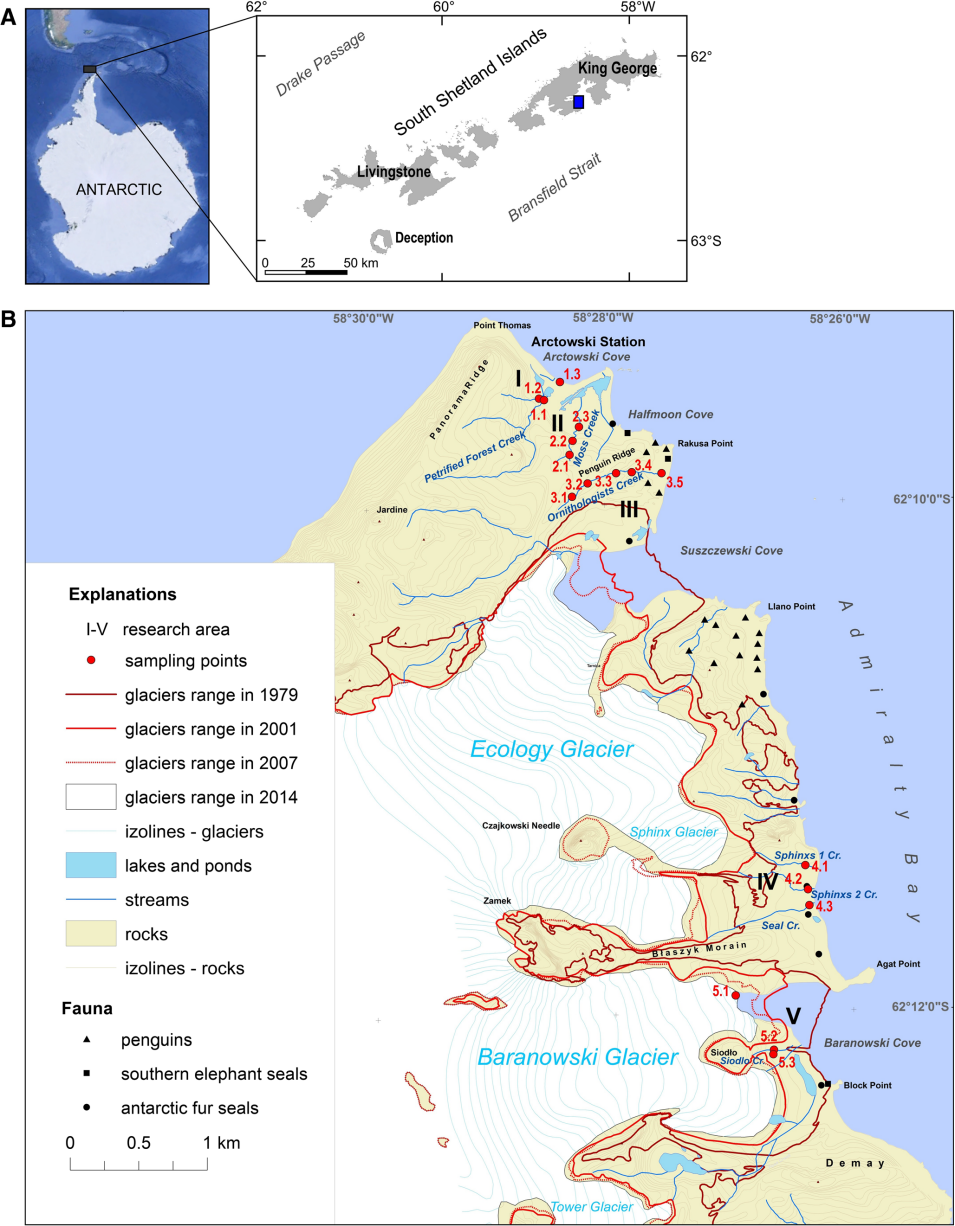
Study area: A, location on the background of the Maritime Antarctica; B, map of the western shore of Admiralty Bay (Maritime Antarctica) showing the location of sampling points and glaciers’ retreats between 1979 and 2014 (prepared based on: Pudełko 2008; Landsat image LC82181032014016LGN00 obtained from www.usgs.gov; GoogleEarth application)

The study area is located on the western shore of Admiralty Bay (King George Island, South Shetland Islands, Antarctic Peninsula, Fig. 1). The research was conducted on eight streams flowing in one of the largest ice-free areas on the western shore of Admiralty Bay (Fig. 1). Water samples were collected from five different sites running from the Arctowski Polish Antarctic Station to the Baranowski Glacier (including creeks from the Antarctic Specially Protected Area (ASPA No. 128). Moreover, a surface snow sample was collected to verify the potential influence of atmospheric deposition on the contamination level. Samples were collected twice: in late January and March 2016. The total amount of selected water and snow samples is presented in Table 1. A basic description of the studied water creeks is presented in the Supplementary Material (Table S1), and a detailed description of the sampling area is available in Szopińska et al. (2018). Water samples (1 L each) were collected manually (using nitrile gloves) without air bubbles to avoid transfer of more volatile compounds to the headspace phase. A surface snow sample was collected manually into a polypropylene container (2 L), also using nitrile gloves. All containers were pre-treated using deionised water (resistivity at 25 °C = 8.2 MΩ cm) to avoid cross-contamination. After collection, all samples were transported to the H. Arctowski Antarctic Station Laboratory and stored frozen at − 20°C, and then transported under unchanged temperature conditions to Poland.

**Table 1:** Detailed description of sample numbers

Sampling area	Petrified Forest Creek	Moss Creek	Ornithologists Creek	Streams near Sphinx Glacier	Streams near Baranowski Glacier	Surface snow near Baranowski Glacier	Σ
Number sampling points	3	3	5	3	2	1	17
Number of samples (January)	3 W	3 W	5 W	3 W	2 W	1 S	16 W + 1S
Number of samples (March)	3 W	3 W	5 W	3 W	2 W	0	16 W
Total amount of samples collected during austral summer 2016	6 W	6 W	10 W	6 W	4 W	1 S	32 W + 1S

*W*, water samples; *S*, snow samples

### Laboratory methods

The main groups of organic compounds in water samples were identified with the use of two-dimensional gas chromatography coupled with time-of-flight mass spectrometry (2D GC-TOF-MS). The GC × GC system consisting of an Agilent 7890A (Agilent Technologies, USA) equipped with a liquid nitrogen-based quad-jet cryogenic modulator and a split/splitless injector, and Pegasus 4D TOFMS (LECO Corp., St. Joseph, MI, USA). The identification of organic compounds was performed by means of comparing the mass spectrum obtained from the experiment against the one included in the NIST 2011 library. In relation to the part of the studies performed with the 2D GC-TOF-MS technique, a duplicate analysis was applied in order to confirm the quality of the results (reproducibility). No quantitative analysis after identification at 2D GC-TOF-MS was performed.

Polycyclic aromatic hydrocarbons (PAHs, ng L^−1^) were identified and quantified with the use of gas chromatography coupled with single quadrupole mass spectrometer (GC-MS)—Agilent 7890A, 5977B MSD system (Agilent Technologies, USA). The determination of selected PAHs was performed based on the internal standard calibration method. Before extraction, surrogate standards have been added. The recovery (%) of individual elements and parameters from reference materials was in the 70–85% range and the recoveries of surrogate standards were 80% and 85% for naphthalene-d8 and benzo(a)anthracene-d12, respectively. Replicate samples and reference solutions were run after every ten samples to assure the precision of each run. Internal standards were used for quantitative analysis. The isotope marking of the internal standards (naphthalene-d8, benzo(a)anthracene-d12) made it possible to calculate the relative response factors (RRF), which help to correct the fluctuations which depend on the conditions of the analysis (Rome and McIntyre 2012). In the case of PAH analysis (GC-MS), measurements of samples were performed in triplicate. After every 10 analyses of the environmental samples, a blank analysis was provided to eliminate potential contamination from the preceding analysis and to verify the proper working order of the chromatograph. All blanks were analysed in the same setup as the samples, using the same reagents, in deionised water featuring levels below the detection limit for each PAH. The exception was naphthalene, for which the concentration in the blank was 0.11 ng L^−1^. In this case, the result obtained in samples was reduced by this value. This way, the background level present in the reagents and analytical containers used during the analytical procedure could be eliminated. The limit of detection for each analyte was from 0.03 to 0.75 ng L^−1^.

A detailed description of all analytical procedures (technical specifications of the equipment, standard solutions and sample preparation methods) are summarised in the Supplementary Material (Table S2).

### Statistical methods and PAH flux calculation method

Principal component analysis (PCA) was employed to describe reveal-hidden dependencies between measured parameters, contaminants concentration and sampling points (a multivariate data set). MATLAB Version: R2013a with Statistics Toolbox Version 9.1 manufactured by MathWorks, USA was used. The approximate average load of ΣPAHs introduced into Admiralty Bay at the beginning of the Southern summer 2016 was calculated for one watercourse (Siodło Creek) within the selected model area 5. For this watercourse, chemical analysis and flow values were available for January 8 to March 23, 2016. The results of flow measurements from January 8 to February 11 were taken from Sziło and Bialik (2017), while flows in Siodło Creek for February 12 to March 23 were calculated on the basis of unpublished flows of Fosa and the relationship between these gauges. Both streams are supplied with ablation water from the Baranowski Glacier and the correlation coefficient between flows in both watercourses for the period January 8 to February 11 was 0.91 (correlation statistically significant for *α* = 0.001).

The average value of ΣPAHs from January and March for the area 5 (the Baranowski Glacier forefield, samples 5.1 and 5.2, Fig. 1) was assumed for the calculation, i.e. *c*_mea*n*_ = 41.6 ± 8.9 ng L^−1^. Then, calculations of the daily load were made (*L*_(24h)_) according to Eq. (1)

1 $$ {L}_{\left(24\mathrm{h}\right)}={c}_{{\mathrm{mean}}^{\ast }}{V}_{\ast }t\left[\mu \mathrm{g}\right] $$

**Fig. 2:**
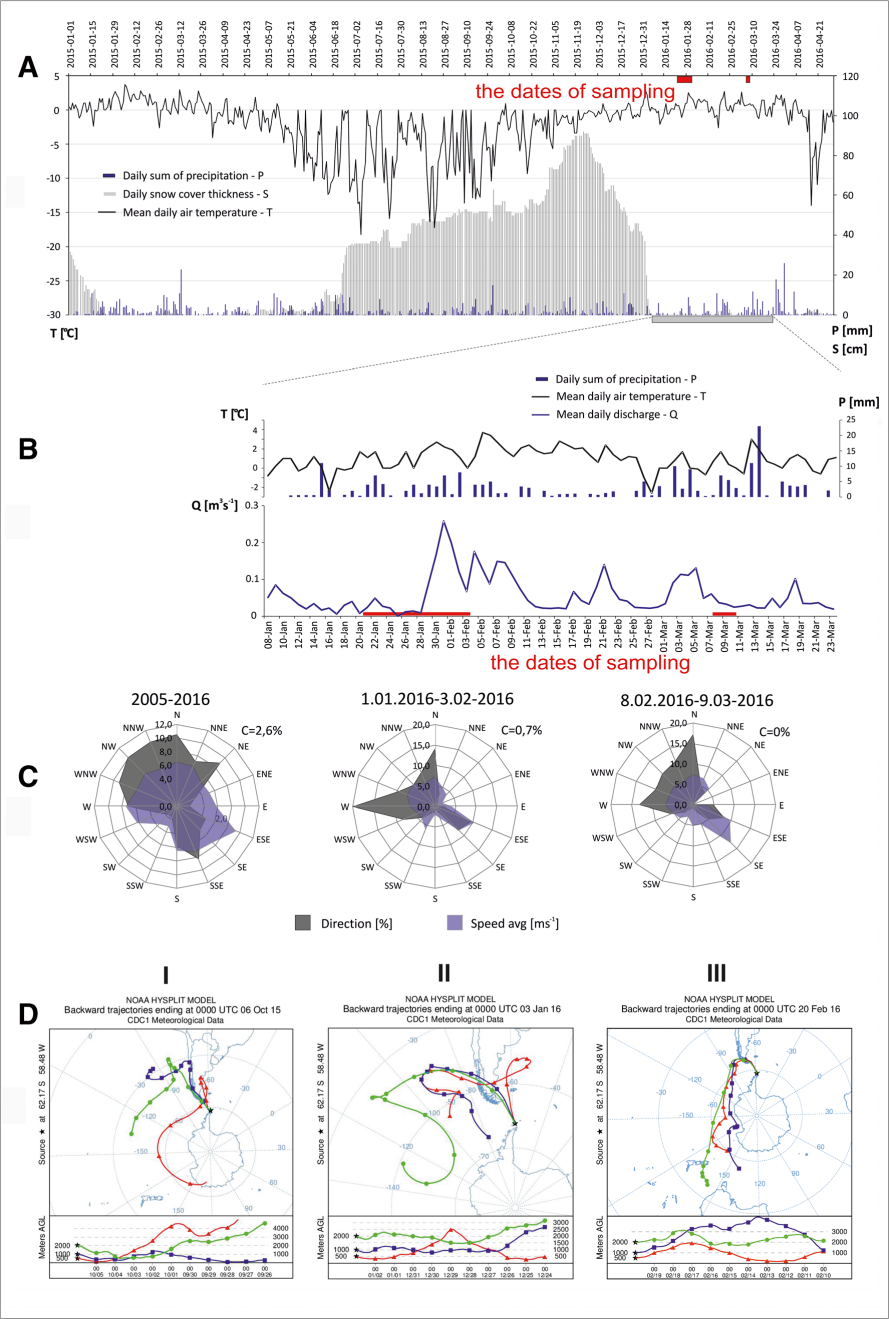
Meteorological conditions on King George Island: **a** weather conditions in the period from January 1, 2015 to April 30, 2016, **b** weather conditions and discharges in Siodlo Creek in the period from January 8 to March 23, 2016, **c** wind directions and wind speed in the period 2005–2016 and during the months preceding sampling (meteorological data based on data available at www.rp5.ru, at the Bellingshausen Station, discharges based on Sziło and Bialik, 2017) and **d** a 10-day backward air mass trajectories (I—from June 10, 2015, II—from March 1, 2016 and III—from October 2, 2016 (computed with HySPLIT based on Global Data Assimilation System meteorological data)

*C*_mean_: mean concentration of the analyte (ng L^−1^)
*V*: flow rate, measured once a day (m^3^ s^-1^)
*t*: time (1 day = 86,400 (s))

taking into account the changing flows rates (Fig. 2b) between January 8 and March 11, 2016. The method of calculation is based on an estimation of load delivered by Siodło Creek during the Southern summer using average values obtained from two sampling dates for this watercourse. Nevertheless, based on this assumption, the obtained estimated load values may represent noteworthy information about the actual PAHs level introduced to the Admiralty Bay and at the same time will constitute baseline values for future analysis and calculations.

## Hydrological and meteorological background

Samples were collected in the Austral summer of 2016 (Fig. 2a). The mean air temperature for 1969–2016 in Bellingshausen Station on the Fildes Peninsula (King George Island) was calculated as − 2.3°C (based on data obtained from www.rp5.ru), and the mean total annual precipitation was 697 mm. The research year 2016 was warmer, with a mean temperature of − 1.8°C, and drier, with total precipitation of 560 mm (Szopińska et al. 2018).

The hydrological condition of creeks with glacial supply is directly related to ablation processes and characterised by rapid outflows (Rachlewicz 1997; Rakusa-Suszczewski 1995; Sziło and Bialik 2017). However, air temperature during the summer season influenced not only glacier ablation, but also the thawing of snow and permafrost (including buried ice), and caused seasonal changes in the creeks’ outflow and the sources of its water (Zwoliński 2007; Szopińska et al. 2018). Moreover, rapid outflow associated with high precipitation and the sudden disappearance of snow cover causes the active erosion of suddenly thawing ground (Rachlewicz 1997). Before sampling, average daily temperatures ranged around 0 °C in January and 1–2 °C in February (Fig. 2a). The highest daily temperature (4.7 °C) was recorded for March 3, and the lowest (4.1 °C) for February 16. There was a thaw in the snow cover towards the end of December 2015 and it rained during the sampling period. Total daily precipitation did not go above 5–10 mm (Fig. 2a). During the researched months, discharges in one sampled creek—the Siodło Creek—were measured (Sziło and Bialik 2017). Flows in the Siodło stream in the period from January 8 to March 23, 2016 fluctuated between 0.006 and 0.26 m^3^ s^−1^ (Fig. 2b), the mean flow value was 0.06 m^3^ s^−1^, while the median was 0.04 m^3^ s^−1^. During the sampling of the watercourse, on January 20 and February 8, similar hydrological conditions prevailed, with low flows at 0.008 and 0.146 m^3^ s^−1^, respectively.

General wind directions at the Bellingshausen Station are presented in Fig. 2b. The predominant wind directions at the Bellingshausen are northwesterly (30%—NNW, NW, WNW), northerly (10%—N), northeasterly (9%—NE) and southeasterly (8%—SSE). The prevailing directions were northerly and westerly in January 2016, and northerly, northwesterly and westerly in February 2016 (in the months preceding sampling). Despite the inflow of pollutants to the study area being limited by the range of circumpolar air circulation, the possibility of long-range air transport of contaminants to the Antarctic has been indicated in some works (Lee et al. 2004; Mishra et al. 2004; Kallenborn et al. 2013). Detailed analysis of a 10-day air mass back trajectories during 24 months, including sampling time (September 2015 to August 2017), shows that air masses came into King George Island mainly from the northwestern and western and that South America was very frequently the sourcing area of air masses (Szumińska et al. 2018). Selected air mass trajectories showing long-range transport of air masses during the sampling period are presented in Fig. 2c.

## Results

### Qualitative characteristics of volatile and semi-volatile compounds present in water samples (2D GC-TOF-MS analysis)

**Fig. 3:**
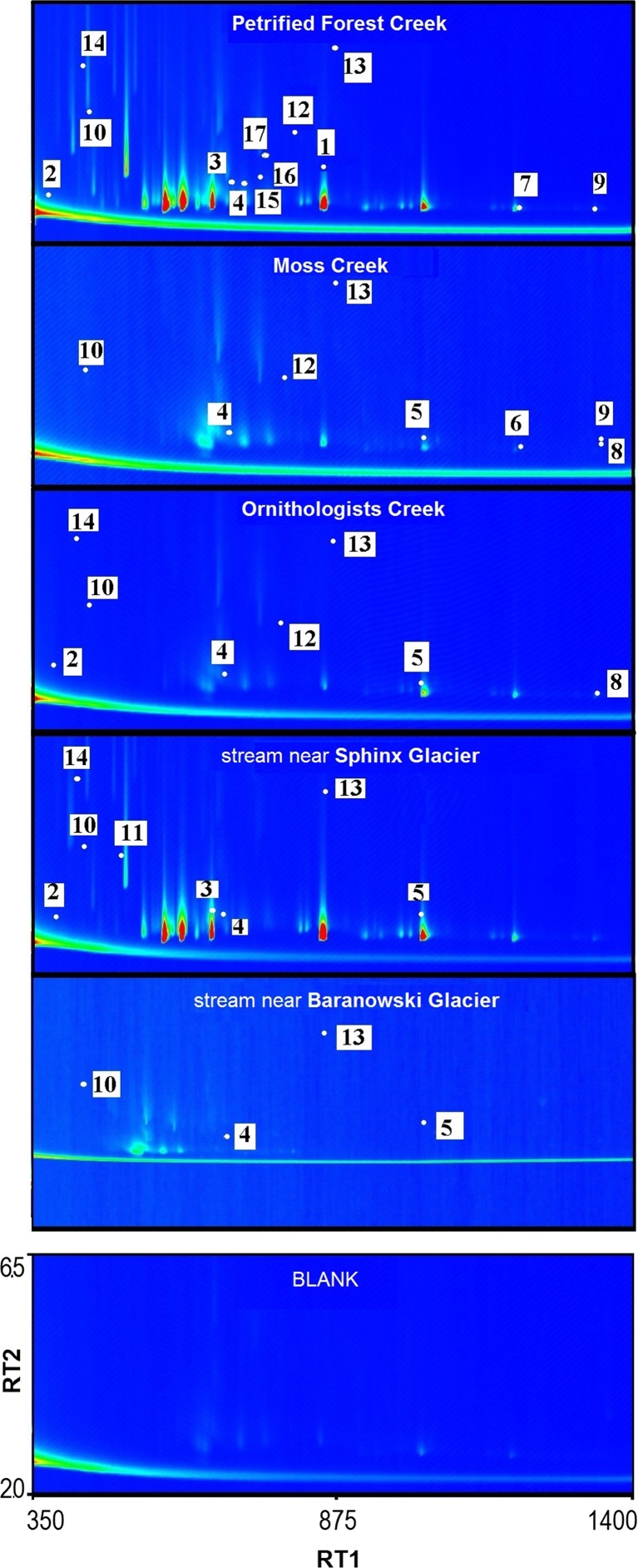
GCxGC–TOF-MS chromatograms of the dichloromethane extracts for selected samples taken in January 2016 from areas I–V. The thermochromic scale expresses intensity. Retention times are given in seconds. Each number presented on chromatograms corresponds to the compound numbers listed in Table 2. RT1, retention time in the first dimension; RT2, retention time in the second dimension

Table 2 lists the identified volatile and semi-volatile compounds. In samples, we can identify compounds belonging to the group of aldehydes, alkanes, aromatics, non-aromatics, PAHs and terpenes. The results in Table 2 show a downward trend in the number of identified volatile and semi-volatile compounds present in samples on the transect from the Arctowski Polish Antarctic Station to the Baranowski Glacier. This trend is also visible on Fig. 3, which shows chromatograms of samples taken in the estuaries of the studied creeks. The samples taken from the Petrified Forest and Moss creeks have the highest number of various alkenes (Table 2).

**Table 2:** List of compounds identified in DCM extracts of water samples taken from the western shore of Admiralty Bay over the different streams in summer 2016

No	Compound name	Chemical class of compound	RT1 (s)	RT2 (s)	Blank sample	Petrified Forest Creek (*n* = 6)	Moss Creek (*n* = 6)	Ornithologists Creek (*n* = 10)	Streams near Sphinx Glacier (*n* = 6)	Streams near Baranowski Glacier (*n* = 4)	Surface snow near Baranowski Glacier (*n* = 1)
1	Decanal	Aldehyde	839	1.716	−	**+**	−	−	−	−	−
2	2,2,4-Trimethylpentane	Alkanes	370	1.426	−	**+**	−	**+**	**+**	−	−
3	2,2,3,3-Tetramethylbutane	Alkanes	649	1.492	−	**+**	−	−	**+**	−	−
4	2,2,4,6,6-pentamethylheptane	Alkanes	659	1.492	−	**+**	**+**	**+**	**+**	**+**	**+**
5	Tetradecane	Alkanes	1018	1.412	−	−	**+**	**+**	**+**	**+**	−
6	Pentadecane	Alkanes	1173	1.426	−	−	**+**	−	−	−	−
7	Hexadecane	Alkanes	1173	1.399	−	**+**	−	−	−	−	−
8	Eicosane	Alkanes	1308	1.426	−	−	**+**	**+**	−	−	−
9	Nonadecane	Alkanes	1308	1.399	−	**+**	**+**	–	−	−	−
10	Toluene	Aromatic	430	1.940	−	−	**+**	**+**	**+**	**+**	−
11	Ethylbenzene	Aromatic	520	1.914	−	−	−	–	**+**	−	−
12	1-Methyl-4-(1-methylethenyl)benzene	Aromatic	739	1.914	−	**+**	**+**	**+**	−	−	−
13	Naphthalene	PAHs	829	2.548	−	**+**	**+**	**+**	**+**	**+**	−
14	1,3,5-Cycloheptatriene	Non-aromatic	425	2.284	−	**+**	−	**+**	**+**	−	−
15	alpha-Ocimene	Terpenes	674	1.518	−	**+**	−	−	−	−	−
16	alpha-Pinene	Terpenes	674	1.505	−	**+**	−	−	−	−	−
17	l-Limonene	Terpenes	689	1.624	−	**+**	−	−	−	−	−

*RT1*, retention time in the first dimension; *RT2*, retention time in the second dimension; *n*, number of samples; “+”, identified in the sample; “−“, not identified

### PAH distribution at the western shore of Admiralty Bay

**Fig. 4:**
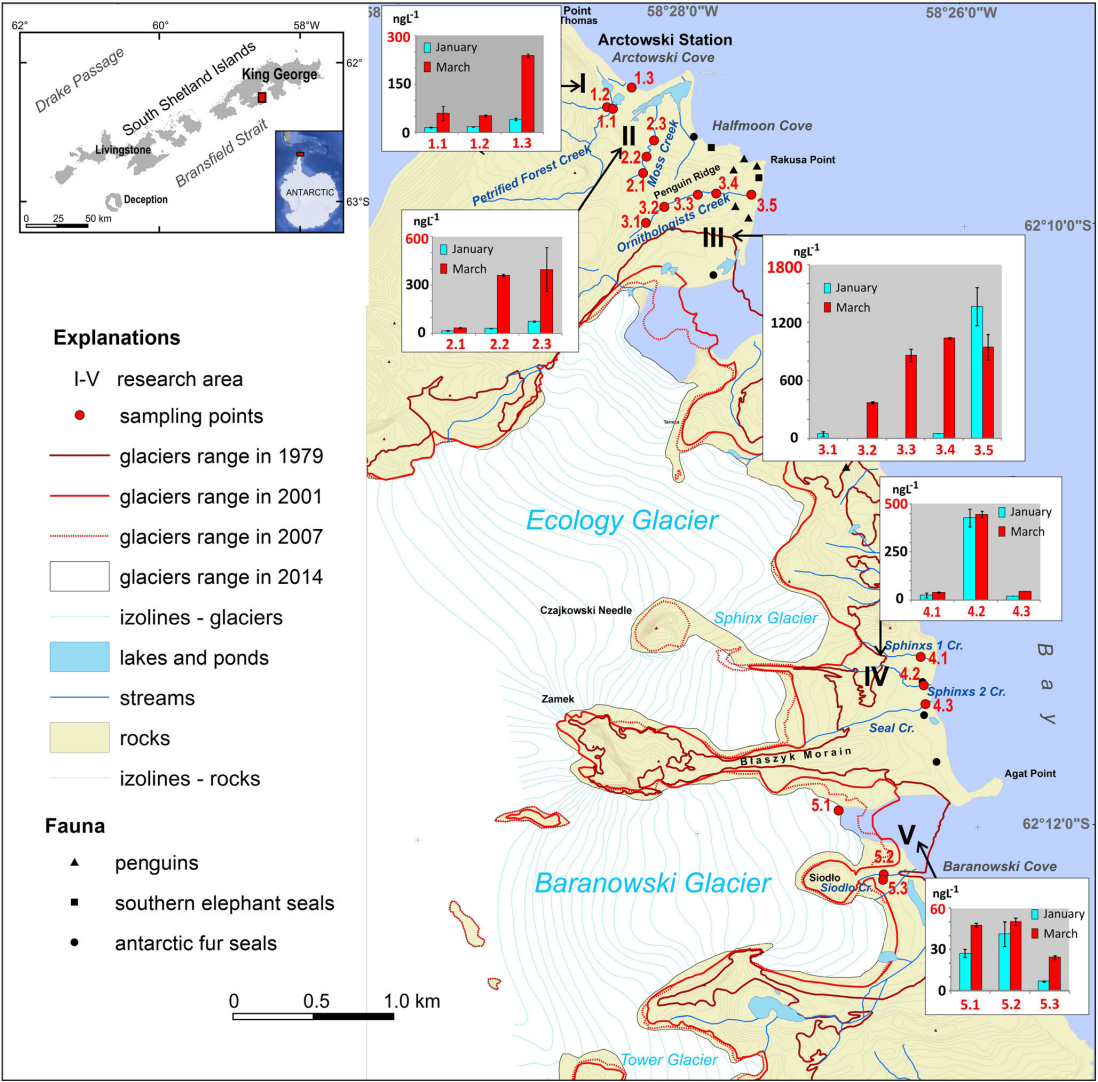
Distribution of total PAHs in freshwater samples during summer 2016. Error bar reflects standard deviation. (Prepared based on author’s analysis and Pudełko 2008; Landsat image LC82181032014016LGN00 obtained from www.usgs.gov; GoogleEarth application)

The results of PAH analytical data analysis are summarised in Table 3. We understand total PAHs (also ΣPAHs) to be the total sum of 16 EPA PAHs. The range of total PAHs is 0.25–1365 ng L^−1^ and 0.28–1037 ng L^−1^ for January and March 2016, respectively (Fig. 4) Low molecular weight (LMW, two- and three-ring PAHs) PAHs such as naphthalene, anthracene and phenanthrene are compounds that have a significant input in the ΣPAHs during both data series. Anthracene, acenaphthene and fluorene are only observed in samples taken in January. High-molecular-weight (HMW, four- and five-ring PAHs) PAHs such as chrysene and benzo(a)anthracene are mostly observed in January (areas I–III and V). The exception is sample 1.3, where chrysene and benzo(a)anthracene are detected in March. What is more, anthracene dominates (61.2–66.7%) in the Petrified Forest and Moss Creek samples nos.: 1.3, 2.1, 2.3 (Table 3). In the case of March data series, there was a dominance of either naphthalene or phenanthrene in all samples in March. Moreover, totally different percentage distribution of PAHs is noted in a snow sample (fluorene, 72.1%; chrysene, 24.4%; benzo(a)anthracene, 3.4%). The absence or negligible concentrations of 5- and 6-ring PAHs (< LOD) were expected results because of their properties (they are non-volatile and less water-soluble than other PAHs) (Jiries et al. 2000). However, it cannot be unambiguously concluded, if those PAHs are completely absent from the samples or merely absent from a performed GC-based analysis. To resolve this, other methods of water sample analysis are recommended to consider (both in terms of sample preparation and determination step).

**Table 3:**
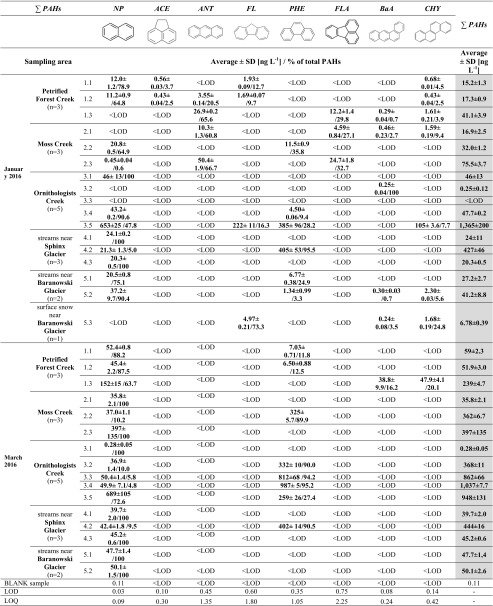
List of PAHs determined in DCM extracts of water samples taken from the western shore of Admiralty Bay over the different streams in summer 2016

*NP*, naphthalene; *ACE*, acenaphthene; *ANT*, anthracene; *FL*, fluorene; *PHE*, phenanthrene; *FLA*, fluoranthene; *PYR*, pyrene; *BaA*, benzo(a)anthracene; *CHY*, chrysene; *LOD*, limit of detection; *LOQ*, limit of quantification; *n*, number of samples

### PAH fluxes introduced into Admiralty Bay during austral summer 2016

The approximate average ΣPAH load introduced in the water phase to Admiralty Bay during the Southern summer is 0.21 g/day. The data relating to the selected model area (the forefield of the Baranowski Glacier), where the most intense effects of glacier retreat in the entire research area are observed.

### PAH sources identification (PAH ratios and PCA)

PAH ratios (also known as PAH diagnostic ratios, PAH indicator ratios) is a well-known tool to differentiate PAH sources between the petrogenic (such as crude oil, fuels and lubricants, and their derivatives) and the pyrogenic (such as oxygen-dependent, high-temperature combustion of fossil fuels and biomass). Despite the availability of a wide range of PAH indicator ratios, described in inter alia Stogiannidis and Laane (2015), based on our results, only 3 PAH diagnostic ratios could be calculated, and these are summarised in Table 4. It is due to the lack of specific PAHs determination results, which are required to other indexes calculation. Identification of sources based on this result is ambiguous (in some cases, indices show a different source). However, in the majority of the results, a petrogenic origin is indicated as the source of PAHs. Therefore, in evaluating the sources, too, the results presented in Table 3 should be examined in detail.

**Table 4:** PAH indicator ratios for research water samples and the potential sources of emission of these compounds

PAHs indicator ratio^a^	NP/PHE	BaA/CHY	ΣLMW/ΣHMW	NP/PHE	BaA/CHY	ΣLMW/ΣHMW
Sampling area	January			March		
1.1	–	–	21.5	7.47	–	–
1.2	–	–	39.9	6.99	–	–
1.3	–	0.18	1.91	–	0.809	1.76
Area I/source	–	Petrogenic	Petrogenic	Pyrogenic	Pyrogenic	Petrogenic
2.1	–	0.29	1.55	–	–	–
2.2	1.80	–	–	0.114	–	–
*2.3*	–	–	2.06	–	–	–
Area II/source	Pyrogenic	Petrogenic	Petrogenic	Petrogenic	–	–
3.1	–	–	–	–	–	–
3.2	–	–	–	0.111	–	–
3.3	–	–	–	0.062	–	–
3.4	9.61	–	–	0.051	–	–
3.5	1.70	–	12.0	2.66*	–	–
Area III/source	Pyrogenic	–	Petrogenic	Petrogenic/*pyrogenic	–	–
4.1	–	–	–	–	–	–
4.2	0.052	–	–	0.106	–	–
4.3	–	–	–	–	–	–
Area IV/source	Petrogenic	–	–	Petrogenic	–	–
5.1	3.04	–	–	–	–	–
5.2	27.8	0.13	14.9	–	–	–
5.3	–	0.14	2.59	–	–	–
Area V/source	Pyrogenic	Petrogenic	Petrogenic	–	–	–
Source						
Pyrogenic (combustion)	> 1^b^	> 0.5–1^c^	< 1^d^	> 1^b^	> 0.5–1^c^	< 1^d^
Petrogenic	< 1^b^	< 0.25–0.5^c^	> 1^d^	< 1^b^	< 0.25–0.5^c^	> 1^d^

^a^*ΣLMW*, sum of low molecular weight PAHs (two- and three-ring PAHs); *ΣHMW*, sum of high molecular weight PAHs (four- and five-ring PAHs); *NP*, naphthalene; *PHE*, phenanthrene; *BaA*, benzo(a)pyrene; *CHY*, chrysene

^b^Ravindra et al. 2008

^c^Stogiannidis and Laane 2015

^d^Zhang et al. 2008

**Fig. 5:**
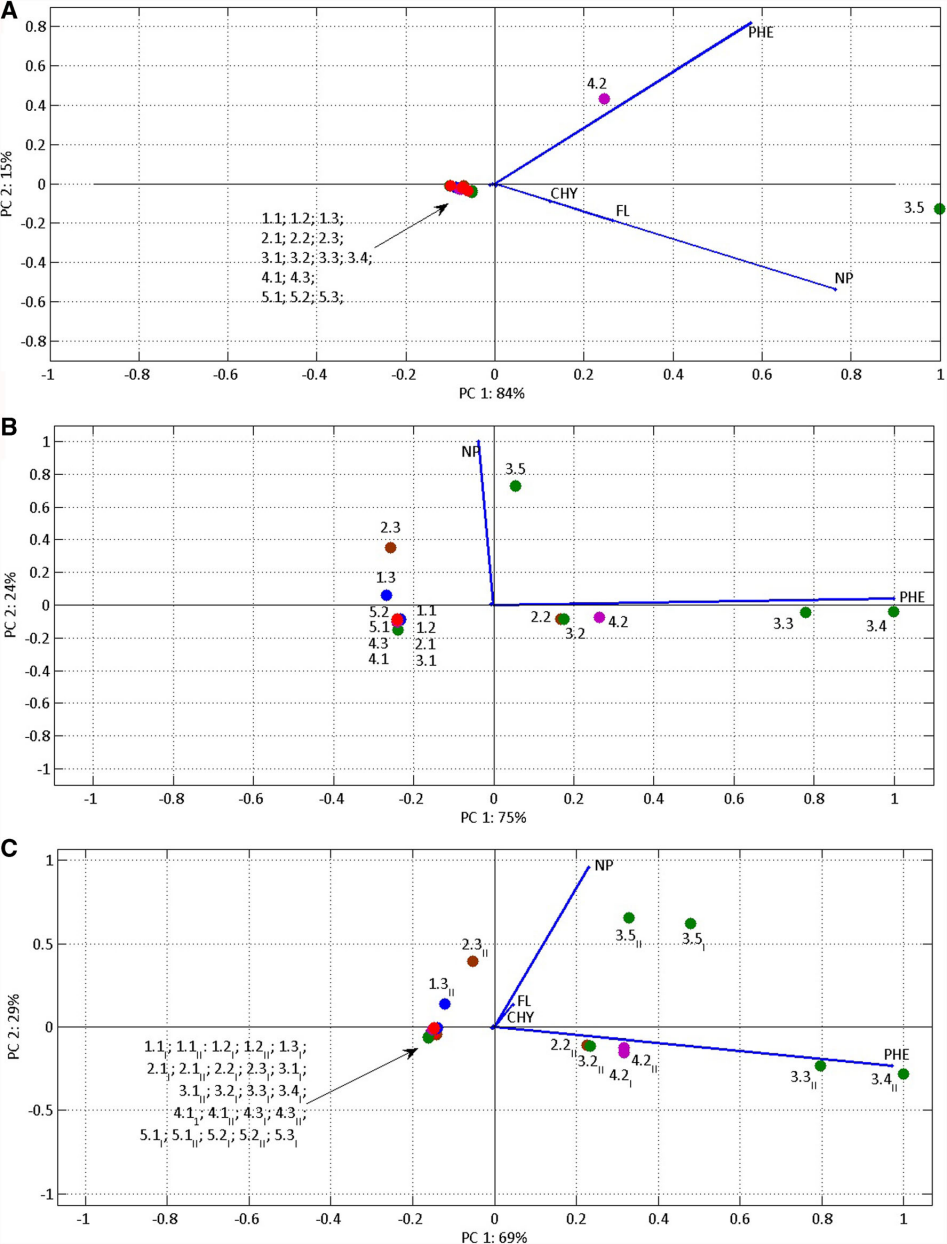
PCA Biplots for various data sets. Projection of environmental variables and cases (sampling points) on the plane of two principal components: **a** data set for January, **b** data set for March, **c** entire data set (January and March)

Moreover, we decided to apply another tool to find out the source of these water components—principal component analysis (Fig. 5). All variables were taken into account for PCA, which was carried out for three cases (series 1, January; series 2, March, the combination of series 1 and 2). Such analysis allowed the possible changes in the water’s organic chemical composition to be demonstrated. For all three series of data, two principal components were identified that represent 99%, 99% and 98% of the variance, respectively, and for all considered cases PC1 and PC2 was found to have a strong correlation with only naphthalene (NP) and phenanthrene (PHE). For the January data set, PC1 was strongly positively correlated with both variables, whereas for the second and entire data, PC1 was strongly positively correlated with PHE, while PC2 was strongly correlated with NP. The analysis of the entire data set shows that fluorene (FL) and chrysene (CHY) are also correlated with PC1 and PC2, but these correlations are much lower than those with NP and PHE.

## Discussion

### Natural factors shaping water chemistry characteristics

Some groups of compounds may be both natural and anthropogenic origin (e.g. PAHs). Even more, many environmental factors may have an influence on their transformation and degradation processes, as well as on their environmental fate (e.g. photochemical degradation, Fasnacht and Blough 2002). Therefore, it is necessary to consider any natural sources of the studied groups of compounds. Considering the natural sources of PAHs, we think about long-range atmospheric transport of volcanic contaminants or forest combustion products: (1) delivered via the atmosphere during the study time (dry and wet deposition) (Fig. 2c), and (2) deposited over hundreds of years in snow/firn (Fuoco et al. 2012). Although a forest fire in South America may not be classified as a natural phenomenon (many such fires are caused by humans—deliberately or accidentally as a result of negligence) (Úbeda and Sarricolea 2016), the received combustion product of biomass will be considered as natural. One should note that the fire events common in underdeveloped areas (Molina et al. 2015) may also influence the chemical status of the atmosphere and these have not been researched before as a source of pollutants in Antarctica. Moreover, some organic contaminants such as short-chain n-alkanes (Table 2) may also be of biological origin (e.g. produced by phytoplanktonic organisms) (Dauner et al. 2015). However, the biogenic sources of PAHs will need to be further investigated as long as the profiles of PAHs derived from the degradation of diterpenes remain unknown (Cabrerizo et al. 2014).

**Fig. 6:**
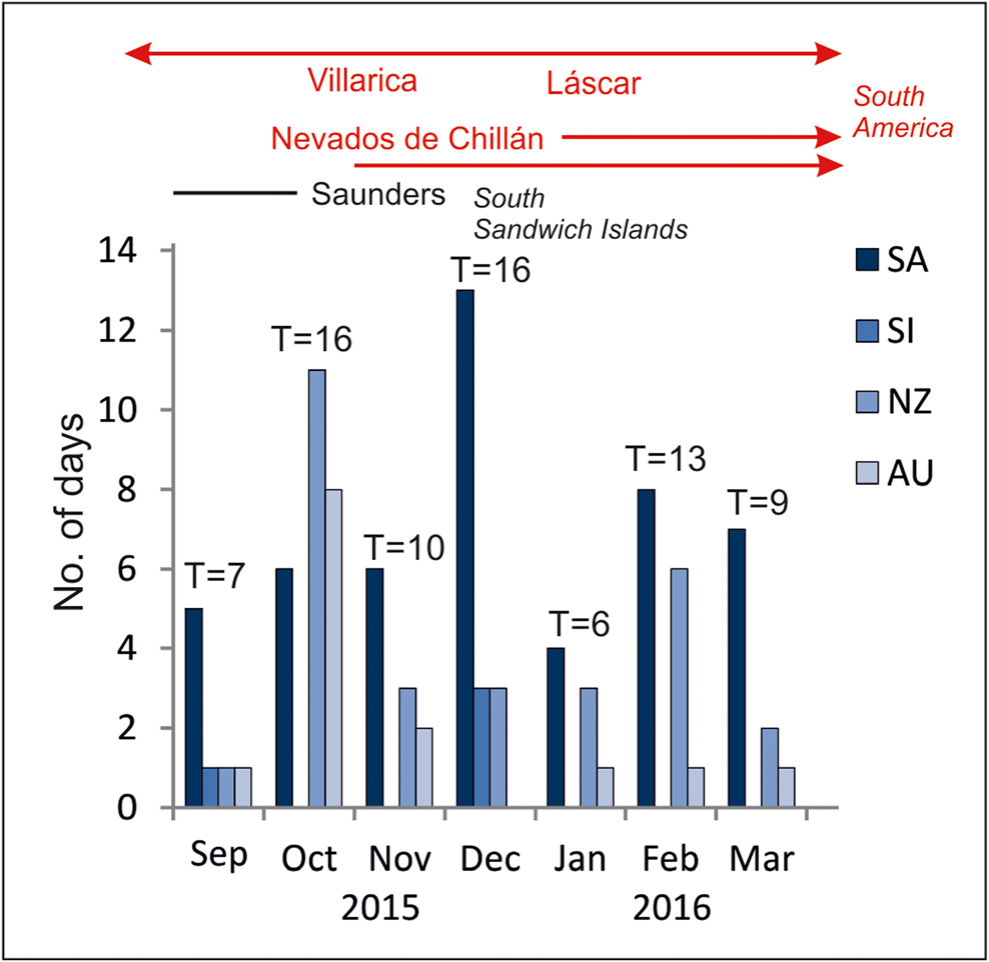
Total number of days with air masses incoming from SA, South America; SI, South Sandwich Islands; NZ, New Zealand; AU, Australia in the particular months during the period of September 1, 2015–March 31, 2016 compared with volcanoes’ activity (after Szumińska et al. 2018, changed)

Some PAHs are considered as being of volcanic origin, e.g. from volcano exhalations (Dauner et al. 2015; Kozak et al. 2017). A research study performed on Spitsbergen (Arctic) after the Icelandic volcanic eruptions of 2010 and 2011 indicates an increase in the concentration of ∑PAHs 695–6797 ng/L and 101–3477 ng/L, respectively. In relation to preceding years 2009, 2012 and 2013, the concentration levels amounted to 4.0–603 ng/L (Polkowska et al. 2011), 13.3–104 ng/L and 59.7–332 ng/L, respectively (Kozak et al. 2017). Moreover, the research on the four-century snow/firn records of polycyclic aromatic hydrocarbons (PAHs) at Talos Dome (Antarctica) showed a very clear correlation between volcanic eruptions and PAH concentration maxima (Fuoco et al. 2012). Fuoco et al. (2012) also point out that the total concentration of the three most abundant non-alkylated PAHs, i.e. phenanthrene (PHE), fluoranthene (FLA) and pyrene (PYR) represents about 70% of ΣPAH and shows no significant change from 1930 to 1980. This helps us to take into consideration that those PAHs present in Antarctic water samples can also be of natural (volcanic) origin. The past South American and New Zealand volcanic eruptions could have affected the chemical composition of air masses, snow, precipitation and, resultantly, also sediments and glacier ice. The number of volcanoes that have been active during the last 500 years were calculated at 7 in Antarctica, 6 in the South Sandwich Islands, 42 in South America and 6 in New Zealand (Szumińska et al. 2018). The most active volcano is Villarica located in central Chile, which activity was recorded in 142 years from 1558 to 2017. In 2015 and 2016, several volcanoes were active in South America, the South Sandwich Islands and Antarctica. Four volcanoes in Chile were still active until 2017: Villarica, Copahue, Láscar, Nevados de Chillán. The analysis of back trajectories shows the possibility that air masses incoming to the South Shetland Islands originated over the active volcanic regions (Szumińska et al. 2018). According to the data presented by the mentioned authors, LRAT occurred most frequently in October and December 2015, taking into account the months preceding sampling for organic pollutants. Furthermore, in January–March 2016, the air masses incoming from South America 19 times and from the South Sandwich Islands 11 times. Air masses originated mainly in the south of the South American continent. However, on October 5th and 6th, air masses originated over the central part of the Andes, where the Villarica volcano was active at the time (Fig. 6). One should note that areas presented in Fig. 6 could be a source area not only of volcanic pollution but also of anthropogenic emission. However, volcanic ash may be delivered to King George Island not only from long distances (Southern Hemisphere), but also from Antarctic volcanoes such as Deception Island (Baker et al. 1975), which is the most active volcano in the South Shetland Islands (over twenty identified eruptions during the past two centuries) (Bartolini et al. 2014). This is mainly due to the strong winds and the unusually low tropopause (8–10 km) in the area (Smellie 1999). Under such a condition, volcanic ash fall deposits (even in historical eruptions) are rapidly displaced over neighbouring islands and the Antarctic continent. All volcanic events may be recorded in a tephra layer (Lee et al. 2007) in a lake or marine sediments, as well as in snow/firn layers (Fuoco et al. 2012). Hence, PAHs accumulated in the snow/firn layer may be released into the environment especially during the spring thaw (the so-called spring pulse). The ‘spring pulse’ phenomenon was observed in January in the Ornithologists Creek estuary during work on our previous study (Szopińska et al. 2018) regarding the determination of inorganic compounds (e.g. metals) in water samples. The same phenomenon is visible in terms of ΣPAH contents because in Ornithologists Creek total PAHs is higher in January than in March. The explanation for this phenomenon would be the same: due to variable topography, snowmelt takes a long time and the process spans the summer. As a result, the mouth section of the stream tends to accumulate not only metals but also organic components, such as PAHs.

In Fig. 4, we see a visible increase in total PAH concentrations in March especially in non-glaciated catchments (I–III). Therefore, taking into account these creeks’ supply in March by precipitation and thawing permafrost (Fig. 2a), and analysis of the air-mass inflow in February and March (Fig. 6), it can be stated that this increase is caused by the transport of compounds from South America and probably contaminants stored in the upper part of soil profiles (the active layer). Based on PAH analysis in soil profiles in the vicinity of Jubany Station, Curtosi et al. (2007) (Potter Cove, KGI) show the highest concentration of PAHs in the lowest part of the active layer (between 150- and 200-cm profile depth) and the prevalence of phenanthrene in the sum of PAHs. Based on 2-year observations of PAHs in the soil, the authors state that PAHs deposited on surface soils due to the rapid association of fine soil particles are transported to bay waters in runoff from snow and ice melt as well as by rain waters. The frequent inflow of air masses from South America in February and March 2016 and the volcanic activity going on at the same time may explain the increased ΣPAHs in March as the process of its adsorption with fine particles and inflow into creeks during precipitation events. However, we should also take into account the previous inflow of PAHs and their migration deeper into soil profiles and hence with groundwater into creeks. Analysis of the NP/PHE and BaA/CHY study results for March in the vicinity of the station (area I) indicates combustion (pyrogenic) as the source. However, in other areas (II–IV), the origin in samples for which index values can be calculated is indicated to be petrogenic. In places where it is impossible to calculate the NP/PHE ratio, the principle and only designated compound from the PAH group is NP (samples 2.1, 2.3, 3.1, 4.1, 4.3, 5.1, 5.2). Due to its high volatility, this suggests that the more likely source of origin for both PHE and NP will be long-range atmospheric transport—LRAT (a global source) (Lammel et al. 2015).

In order to obtain some references for global migration of PAHs, a snow sample was collected in January. Analysis showed a predominance of fluorene (72.11%), with a lesser contribution of chrysene (42.52%) and benzo(a)anthracene (3.44%). A slightly different result was obtained by (Na et al. 2011). In samples of snow from the Fildes Peninsula, the principal component analysis revealed that naphthalene, fluorene and phenanthrene were the three main factors of PAHs, accounting for 61%, 22% and 10%, respectively (Na et al. 2011). Based on air mass trajectory analysis (Szumińska et al. 2018), we observe that delivery of potential contamination load may be from different sites in the Southern Hemisphere. To differentiate the sources of each component in detail, more frequent samplings in this area are recommended.

### Anthropogenic factors shaping water chemistry characteristics

Despite its pristine environment, scientific, logistic and touristic activities in the South Shetland Island Archipelago have introduced anthropogenic compounds. Those activities are related to oil storage, fuel combustion and human waste production. The use of fuel is the main source introducing hydrocarbons into the environment (Martins et al. 2004). Bicego et al. (2009) also pointed out that the principal Antarctic sources of PAHs are the direct input of fossil fuels and inputs associated with incomplete combustion. Arctic Grade Diesel Fuel is the principal source of energy in Antarctic operations and it consists mainly of semi-volatile aromatic hydrocarbons, including naphthalene and various other non-substituted PAHs containing two or three aromatic rings (Kennicutt et al. 1991). However, Arctic Diesel Fuel also has a higher level of complex compounds featuring four or more rings.

PAH ratio analysis shows the predominance of petrogenic sources. Taking into consideration the ratio (> 1) from ΣLMW PAHs to ΣHMW PAHs (Table 4), all received results (mainly in January) indicate petrogenic sources, which are anthropogenic in origin (crude oil, fuel, diesel, etc.). This may be interpreted as a result of scientific station activities in the whole South Shetland Island Archipelago. The number of scientific stations (year-round and temporary) is 20 (Bartolini et al. 2014). Furthermore, PAHs isomer pair ratio (BaA/CHY) analysis (Table 4) showed that the major sources of high molecular weight PAHs in January are petrogenic too—fossil fuel/petroleum (gasoline and diesel) (BaA/CHY < 0.5). The gasoline and diesel are used in generators at research stations and as fuel for boats. Moreover, heavier fuels such as kerosene (used for helicopters) are used by different scientific groups within the vicinity of the Arctowski Polish Antarctic Station and other sites around Admiralty Bay. The isomer pair PAHs ratios showed that the contribution of these sources is more evident close to the station than in the other studied areas. This may be related to the beginning of the summer research season and the related, intensive activities of the researchers. Later, in March, the calculation of BaA/CHY ratio was possible only for one sample from Petrified Forest Creek and showed a pyrogenic origin for these compounds. This may instead indicate the global or mixed origin of these compounds. The influence of both local and global sources of PAHs is also visible in results obtained by Martins et al. (2010) based on analysis of sea sediments from Admiralty Bay.

It is interesting that in the vicinity of the Sphinx Glacier, the source is indicated by NP/PHE analysis to be petrogenic. This relates to one sampling site—4.2—where in both January and March, the sum of PAHs is over 400 ng L^−1^. This is a specially protected area where human activity is negligible. Furthermore, such high concentrations of PAHs have not been identified in nearby streams. Field observation shows that the supply of this stream is uneven (being both glacial and from snowflake melt), so this result also would indicate a potential local fuel spill. In this case, PAHs may be reemitted from the soil and sediments and introduced to water. Due to their average volatility, PAH compounds have a tendency to be adsorbed to particulate matter (Abdel-Shafy and Mansour 2016). In addition, the harsh Antarctic climate significantly slows the degradation processes of these compounds (Corsolini 2009), so it is possible that they are being continually flushed out of contaminated soil/surface sediments into the environment (Curtosi 2007).

Short chain n-alkanes (<*C*  20) were detected in all studied areas (Table 2), which we suggest is a result of diesel usage (anthropogenic local source). In the literature, there is a lack of data on the presence of n-alkanes in Antarctic freshwater samples. However, a previous study of geochemical markers of pollution in soil and terrestrial sediments from this area indicates that the distribution of inter alia n-alkanes mostly indicates that the organic matter present in sediments originates from petroleum product spills rather than from the Antarctic environment (Prus et al. 2015). In their studies, sediments with relatively recent contamination recognised near Arctowski Polish Antarctic Station was the richest in short-chain n-alkanes. Martins et al. (2004) also point out that a short chain n-alkanes sequence presence in marine sediments (C12–C21) is associated with Arctic diesel fuel being present in the samples. In turn, aromatic compounds such as toluene, ethylbenzene and any methyl-substituted forms of benzene present in water samples are originally abundant in most crude oils and their presence in the environment is particularly important due to their toxic and carcinogenic properties (Mehlman 2006).

### Source apportionments

Based on our results, and the short discussion described above, it is not possible to clearly indicate a single source (natural or anthropogenic). A similar conclusion was drawn by, inter alia, Cabrerizo et al. (2016) and Bicego et al. (2009). Air mass trajectory analysis suggests the possibility of anthropogenic pollutants having been transported from industrial areas shortly before the samples were collected. However, as was mentioned above, atmospheric transport should also be analysed in the context of natural origins of contaminants, e.g. contemporary and past volcanic eruptions. Taking into account these uncertainties, it would be preferable to try to differentiate sources between long-range (global) and local atmospheric transport, rather between than anthropogenic and natural. However, the significant anthropogenic contribution and threat to the PAH contents in Antarctica should not be forgotten.

A different origin for each of the PAHs during January and March is confirmed by PCA analysis, which shows the predominant importance of NP and PHE, but with different correlations for PC1 and PC2 (in January data set (Fig. 5a) PC1 was strongly positively correlated with both variables NP and PHE, whereas for the March data (Fig. 5b), PC1 was strongly positively correlated with PHE, while PC2 was strongly correlated with NP).

Based on data in the literature, some examples of actual levels of PAHs in different parts of the environment on the South Shetland Island Archipelago and other places in Antarctica are presented in Table 5. The table also presents potential sources of compounds and shows a differentiation between local and global, as well as anthropogenic and natural pollution sources. Considered among the natural sources are, inter alia, PAHs synthesised by bacteria, plants and fungi (McElroy et al. 1989) and volcanic ash (Wilcke 2007), forest fires (Vergnoux et al. 2011). In the case of anthropogenic sources, Table 4 includes industry activities in Southern Hemisphere (Salamanca et al. 2016), oil spills (Aislabie et al. 2004) and scientific station activity (Bargagli 2008).

**Table 5:** Example concentration ranges and sources of PAHs in different samples from the Antarctic (mainly the South Shetland Archipelago region)

Sample type	Localisation	ΣPAHs concentration	Sources of PAHs				LIT.
			Global	Local	Natural	Anthropogenic	
Air (aerosol particles)	Terra Nova Bay	0.014–0.689 ng m^−3^	X			X	Caricchia et al. 1995
Snow/firn	Talos Dome	0.7–4.6 ng L^−1^	X		X	X	Fuoco et al. 2012
Runoff water	Western shore of Admiralty Bay	0.06–1037 ng L^−1^	X	X		X	present study
Soil	Potter Peninsula, Jubany Station, KGI	10–1182 ng g^−1^ dry wt.		X		X	Curtosi et al. 2007
	South Shetland Archipelago (Livingstone and Byers Peninsula, Deception, Barrientos and Penguin Island)	4–99 ng g^−1^ dry wt.	X	X	X	X	Cabrerizo et al. 2016
Suspended particulate matter in marine water	Potter Cove, Admiralty Bay, KGI	31.5–310.9 ng g^−1^ dry wt		X		X	Curtosi et al. 2009
Marine water	Admiralty Bay, KGI	80 ng L^−1^		X		X	Bicego et al. 1996
Marine sediments	Potter Peninsula, Jubany Station, KGI	28–1908 ng g^−1^ dry wt.		X		X	Curtosi, et al. 2007
	Admiralty Bay, KGI	< 6–454.9 ng g^−1^ dry wt.		X		X	Martins et al. 2010
	Prydz Bay	12.95–30.93 ng g^−1^ dry wt.	X				Xue et al. 2016
	Admiralty Bay, KGI	0.71–234 ng g^−1^ dry wt.		X	X	X	Bicego et al. 1996
Tissues from marine organisms (bivalves)	Potter Cove, Admiralty Bay, KGI	80.43–257 ng g^−1^ dry wt.		X		X	Curtosi et al. 2009
Penguins (Gentoo, Chinstrap, Adélie)	Admiralty Bay, KGI	60.1–238.7 ng g^−1^ wet wt		X		X	Montone et al. 2016
Vegetation (mosses)	Admiralty Bay, KGI	121–1235 ng g^−1^ dw		X		X	Colabuono et al. 2015

This short analysis (Table 5) shows that the authors predominantly indicate local anthropogenic sources as being responsible for contamination in this area. However, global sources (long-range atmospheric transport) also influence the presence of such a ubiquitous group of compounds. The least known are the natural sources. Our preliminary research based on one Austral summer season shows a mix contribution of global and local.

Marine and lake sediments provide information on changes in PAH concentrations over decades (Martins et al. 2010), but compounds present in runoff water relatively quick reflect the direct influence of (1) wet and dry deposition (long/short-range atmospheric transport) during the sampling period, (2) release of organic compounds from melting glaciers and (3) potential leaching of adsorbed substances from soil and vegetation. Detailed study of water samples reflecting the dissolved phase of organic compounds may provide a useful tool to differentiate between sources during each season. However, our study indicates that this approach calls for frequent sampling and combining the result together with PAHs in soil, sediment, snow, air or precipitation and vegetation samples.

### The introduction of PAH loads into Admiralty Bay

PAH compounds are relatively easily adsorbed onto solid particles and accumulate in sedimentation environments (bottom sediments, lake sediments) (Curtosi et al. 2007; Abdel-Shafy and Mansour 2016). For this reason also, despite the comparatively low concentrations determined in flowing waters, the total daily load of 0.21 g/day introduced into Admiralty Bay during the Southern summer certainly could cause an increase in the concentrations of these compounds in the bay, and consequently lead to their accumulation in bottom sediments. Analysis of PAHs in sea sediment cores in Admiralty Bay shows different concentrations at particular core depths, with maximum 454.9 ng g^−1^ (of dry weight) occurring at a depth of 3.5 cm in the sediments dated to 1995/1997 (Martins et al. 2010). PAH compounds and other hydrocarbons introduced into the bay may pose a threat to benthic organisms, negatively affecting their development (Curtosi et al. 2009)*.* PAHs have toxic properties and may be harmful to the environment, especially in the case of chronic exposure (Moodley et al. 2017). The 16 PAHs listed as priority pollutants by the United States Environmental Protection Agency (US EPA) are known for their adverse effects on human health (Zhang and Tao 2009) and have long been considered an environmental concern as some of the PAHs are known or suspected to be carcinogenic (Straif et al. 2006). Our result show that NP represents the greatest contribution in chemical composition across the study area (Fig. 5). Naphthalene has been classified as a Group 2B carcinogen (or hazard) by the International Agency for Research on Cancer (IARC) (http://monographs.iarc.fr/ENG/Classification/latest_classif.php, 10 November 2017), which means that it is possibly carcinogenic to humans and animals.

The presence of these compounds in the abiotic environment of Antarctica may be a threat to local fauna and flora. PAHs were detected in penguins and predominantly comprised two- and three-aromatic-ring compounds (Montone et al. 2016) and the same trend in PAHs distribution is seen in water samples during Austral summer 2016 (Fig. 4). It shows that water-soluble PAHs may be bioavailable for living organisms. Moreover, taking into consideration that the study area belongs to ASPA No. 128, there is not so much a need as an obligation to monitor and reduce PAHs and other hydrocarbon levels in this area. We hope that improved fossil fuel economy, and not only on the local scale, would help reduce PAH levels at the western shore of Admiralty Bay, and in the bay itself.

## Conclusions

The analysis of results obtained during the 2016 Austral summer allows the following to be concluded:

1. The presence of n-alkanes and other hydrocarbons (e.g. toluene, ethylbenzene) clearly shows diesel usage as a source (anthropogenic, local source). The PAHs indicator ratio ΣLMW/ΣHMW also indicates petrogenic sources. We believe that improved management of fuel would lead to a rapid decrease in the presence of anthropogenic hydrocarbons (and PAHs) in Antarctica.
2. The increase in total PAH concentration in March 2016 shows long-range atmospheric transport from South America as its source (global sources), and also the possibility of release of PAHs from the active layer.
3. PCA shows differentiation between PAHs’ origin in January and March and a predominance of NP and PHE in water samples during the study period. Both compounds are low molecular weight PAHs, have great volatility and are relatively soluble in water (in relation to other PAHs). Their presence may negatively influence indigenous species (e.g. penguins).
4. The daily load of PAHs dissolved in water introduced into Admiralty Bay in the area of the Baranowski Glacier (Siodło Creek) during the Southern summer was on average 0.21 g/day and may lead to an increase in the concentration of these compounds in the bay waters over the years, and consequently lead to them accumulating in bottom sediments.

Only a few of published studies have considered the long-range atmospheric transport of pollution as a source of PAHs on the King George Island. Even fewer have taken into account natural (volcanic and biogenic) sources. Our analysis indicates the mixed origins of PAHs (global and local). It was not possible to determine the relative shares of individual sources based on data from one Austral summer. Hence, frequent sampling during summer and collecting data are called for over a period of a few years. Analysis of air or precipitation, soil, sediments, snow and vegetation samples should also be conducted. Comparison of the presence of PAHs in different kind of samples would definitely help to differentiate the sources between local and global as well as anthropogenic and natural. Moreover, the potential negative effect of the presence of PAHs and other hydrocarbons in the water environment should be investigated in detail during further research.

## Electronic Supplementary Material

ESM 1 (DOCX 23 kb)
