# Supplementary material for: Determination of polycyclic aromatic hydrocarbons (PAHs) and other organic pollutants in freshwaters on the western shore of Admiralty Bay (King George Island, Maritime Antarctica)
Source: Environ Sci Pollut Res Int. 2019 Apr 29;26(18):18143–61. doi: 10.1007/s11356-019-05045-w (PMC6570687; doi:10.1007/s11356-019-05045-w)
Supplement: Supplementary file 1 — (DOCX 23 kb) [file 11356_2019_5045_MOESM1_ESM.docx]

**Supplementary Material** for

**Determination of polycyclic aromatic hydrocarbons (PAHs) and other organic pollutants in fresh waters on the western shore of Admiralty Bay (King George Island, Maritime Antarctica)**

Małgorzata Szopińska^1^, Danuta Szumińska^2^, Robert Józef Bialik^3^, Tomasz Dymerski^4^, Erwin Rosenberg^5^, Żaneta Polkowska^4*^

1. Gdansk University of Technology, Faculty of Civil and Environmental Engineering, Department of Water and Waste Water Technology , 11/12 Narutowicza St., Gdansk 80-233, Poland
2. Kazimierz Wielki University, Institute of Geography, 8 Kościelecki Sq., 85-033, Bydgoszcz, Poland
3. Institute of Biochemistry and Biophysics, Polish Academy of Science, Pawińskiego 5a, 02-106 Warsaw, Poland
4. Gdansk University of Technology, Faculty of Chemistry, Department of Analytical Chemistry, 11/12 Narutowicza St., 80-233 Gdańsk, Poland;
5. Institute of Chemical Technologies and Analytics, Vienna University of Technology; Getreidemarkt 9/164 AC; A-1060 Vienna, Austria

***** corresponding author: zanpolko@pg.edu.pl

**Main contents:**

**Table S1** Basic description of study area…………………………………………………………………………………………………S2

**Table S2.** Detailed information about analytical procedures, measurement condition, and equipment used in chemical analysis………S3

**Table S1** Basic description of study area

| 1. **Sampling area** | **Names of studied Creeks** | **Length**  **[km]** | **Factors possibly influencing water courses** | **Protection type** |
| --- | --- | --- | --- | --- |
| **I/**  **Petrified Forest Creek** | Petrified Forest Creek | 1.62 | scientific station, possible permafrost occurrence | - |
| **II/**  **Moss Creek** | Moss Creek | 0.91 | scientific station, possible permafrost occurrence, mosses | - |
| **III/**  **Ornithologists Creek** | Ornithologists Creek | 0.90 | possible permafrost occurrence, penguins colony and associated with them ornithogenic soils, mosses in the surface area of the outflow section | ASPA 128 |
| **IV/**  **streams near Sphinx Glacier** | Sphinx 1 Creek | 0.51 | large share of glacier meltwater in outflow | ASPA 128 |
|  | Sphinx 2 Creek | 0.65 | large share of glacier meltwater in outflow | ASPA 128 |
|  | Seal Creek | 0.85 | large share of glacier meltwater in outflow, occurrence of seals | ASPA 128 |
| **V/**  **streams near Baranowski Glacier** | Siodlo Creek | 0.31 | large share of glacier meltwater in outflow | ASPA 128 |
| **Abbreviation:** ASPA 128 – Antarctic Specially Protected Area No. 128 | | | | |

| **Table S2.** Detailed information about analytical procedures, measurement condition, and equipment used in chemical analysis. | | | | | |
| --- | --- | --- | --- | --- | --- |
| **Compounds/**  **Parameters** | **Analytical Procedure/**  **sample preparation method** | **Compounds identification method** | **Standard Solution** | **Instrument** | **Measurement Information** |
| **Volatile and semi-volatile organic compounds** | A 0.5 L sample of water was extracted with 30 mL (2×15 mL) dichloromethane and shaken twice for 20-30 min. After liquid-liquid extraction (LLE), the extract was evaporated to a volume of 300 µL under a gentle stream of nitrogen. | The mass spectrometer was operated in the SCAN mode. Identification of peaks observed in the chromatograms for DCM water samples extracts was made by comparison of the experimental m/z value for the molecular ion with the calculated ones for candidate compounds, resulting from NIST 2011 data base search, with matches above 90%. | - | Instrument: GC × GC system based on 6890A gas chromatograph (Agilent Technologies) and Pegasus IV time-of-flight mass spectrometer (LECO Corp.);  Capillary columns: primary column - Equity 1 (30 m x 0.25 mm x 0.25 µm; 100% Dimethylpolysiloxane),  Secondary column - SolGel-Wax (10 m x 0.10 mm x 0.10 µm; 100% Polyethylene glycol) | Temperature program: initial temperature of 40 °C 1 min, from 40^o^C to 250°C at 10°C min^-1^, maintained for 10 min. The optimized temperature program for the secondary GC oven involved a shift of +5°C in relation to the program of the primary GC oven.  A modulation period of 5 s was employed with the cryogenic trap cooled to -196°C using liquid nitrogen.  Carrier gas: helium, flow of 1.0 mL min^-1^.  Injection mode: splitless  Glass insert: splitless insert with wool  Injection temperature: 250^o^C  Ions in the m/z 40 - 400 range were analyzed with a data acquisition rate of 125 spectra s ^-1^. |
| **PAHs**  Naphthalene  Acenaphthylene  Acenaphthene  Fluorene  Phenanthrene  Anthracene  Fluoranthene  Pyrene  Chrysene  Benzo(b)fluoranthene  Benzo(k)fluoranthene  Benzo(a)pyrene  Benzo(a)anthracene  Indeno(1,2,3-cd)pyrene  Dibenz(a,h)anthracene  Benzo(g,h,i)perylen | A 0.5 L sample of water was extracted with 30mL (2×15 mL) dichloromethane, two deuterated internal standards (naphthalene-d8 (m/z 136) and benzo(a)anthracene-d12 (m/z 240)) were added and shaken for 20-30 min. After liquid-liquid extraction (LLE), the extract was evaporated to a volume of 300 µL under a gentle stream of nitrogen | The mass spectrometer was operated in the selected ion monitoring (SIM) mode. The following ions were monitored (m/z):128,127,152,151,154,153,166,165,178,176,203,202,228,226,252,250,277,276,279. The PAHs were identified and quantified with respect to two deuterated internal standards — naphthalene-d8 (136 m/z) and benzo(a)anthracene-d12 (240, 239 m/z) as well as to the standard PAH solution. | Surrogate standards: deutered naphthalene-d8 and deutered benzo(a)anthracene-d12 (2000µg mL^-1^);  Standard Mixture: QTM PAH mix (2000µg mL^-1^ in methylene chloride; Supplier USA); | Instrument: GC-MS - Agilent 7890A system (Agilent Technologies, USA).  Capillary column: A ZB-5MS (30 m x 0.25 mm x 0.25 µm; 5% Phenyl-Arylene, 95% Dimethylpolysiloxane) | Temperature program: initial temperature 40^o^C, 40^o^C to 120^o^C at 40^o^C min^-1^, then 120^o^C up to 280^o^C at 5^o^C min^-1^, where it was held for 17 min. Carrier gas: helium, flow of 1.5 mL min^-1^.  Glass insert: splitless insert with wool  Injection mode: splitless  Injection temperature: 280^o^C  Injection volume: 2 µL |
| **Total Organic Carbon** | - | - | Potassium Hydrogen Phthalate, C_6_H_4_(COOK)(COOH) Nacalai Tesque, Kyoto, Japan | Instrument: Total Organic Carbon Analyzer  TOC-V CSH (Shimadzu) | Sample injection volume: 150 µL  Measurement method: wet oxidation (680ºC combustion catalytic oxidation)/ non dispersive infrared detection (NDIR) |
